# Magnetic Moment and Spin-State Transitions in Twisted Graphene Nanostructures

**DOI:** 10.1021/acs.jpclett.4c03542

**Published:** 2025-02-18

**Authors:** F. N. N. Pansini, F. A. L. de Souza, V. C. Mota, Wendel S. Paz

**Affiliations:** † Departamento de Física, Universidade Federal do Espírito Santo, Vitória, 29075-910, Brazil; ‡ Instituto Federal de Educação, Ciência e Tecnologia do Espírito Santo, Ibatiba, 29395-000, Brazil

## Abstract

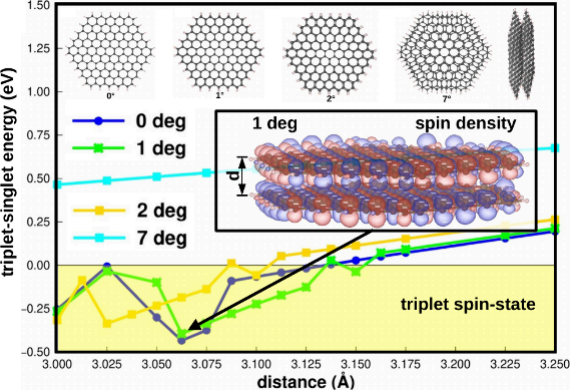

The emergence of magnetic moments and spin-state transitions in the AA-stacking regions of twisted graphene nanoflakes is analyzed by using Density Functional Theory (DFT). Systems of different sizes (C_192_H_48_, C_300_H_60_, and C_432_H_72_) are employed to model some possible stacking angles. Potential Energy Curves (PECs) are computed for different interlayer distances and twist angles, revealing that the triplet ground state appears only in the repulsive region of the PEC, with the transition distance depending on the flake size. The results indicate that interlayer repulsion and twisted angle play significant roles in determining magnetic properties, while spin density analysis confirms that edge effects and AB-region confinement are fundamental to the emergence of magnetic moments in twisted graphene bilayers.

The perspective of manipulating the electron-spin states and localized magnetic moments in nanostructures such as graphene monolayers and nanoflakes is a promising route for the development of applications in classical and quantum computing, as well as quantum communication technologies.^1^ For decades, the experimental investigation of these systems has been restricted by their high chemical reactivity, hindering analyses to ensemble measurements by means of paramagnetic resonance.^2^ The emergence of on-surface synthesis, combined with surface scanning probe techniques, has revolutionized the study of the magnetic properties of these intriguing systems.^3−6^ In this regard, although a pristine graphene monolayer is a nonmagnetic^7,8^ material, magnetic moments may be induced through doping, defects, or surface decoration.^9−14^ Additionally, Lieb’s theorem^15,16^ establishes that the magnetic moments of graphene nanoflakes can be controlled by altering its sizes and shapes, which would meddle with the balance between the number of sites in the sublattices A and B.^17−27^ These structures exhibit magnetic properties described by spin models that reveal exotic features such as spin fractionalization and symmetry-protected topological order, unlocking new opportunities for technological applications and advancements in quantum material science.^28,29^

On the one hand, functionalization, defects, doping, and adding atoms are required for the emergence of magnetism in graphene monolayers,^30−33^ which is essentially associated with the local break of the electronic symmetry in the vicinity of the new bonds. On the other hand, when two graphene sheets are stacked, forming a bilayer, with a rotation angle of approximately 1°, a moiré pattern is created, with both AA and AB stacking regions, as illustrated in Figure 1. The electronic structure of the material is henceforth altered, leading to the emergence of new properties.^34−45^ Specifically, a ferromagnetic coupling arises between the AA stacking regions by only choosing a given magic angle.^39^Figure 1c–e illustrates that, as the twist angle increases, the area fraction of AA stacking regions decreases, whereas that of AB stacking regions increases. This geometrical relationship affects the electronic structure of the systems, altering the conditions for the emergence of the magnetic moment, as discussed in this work.

**Figure 1:**
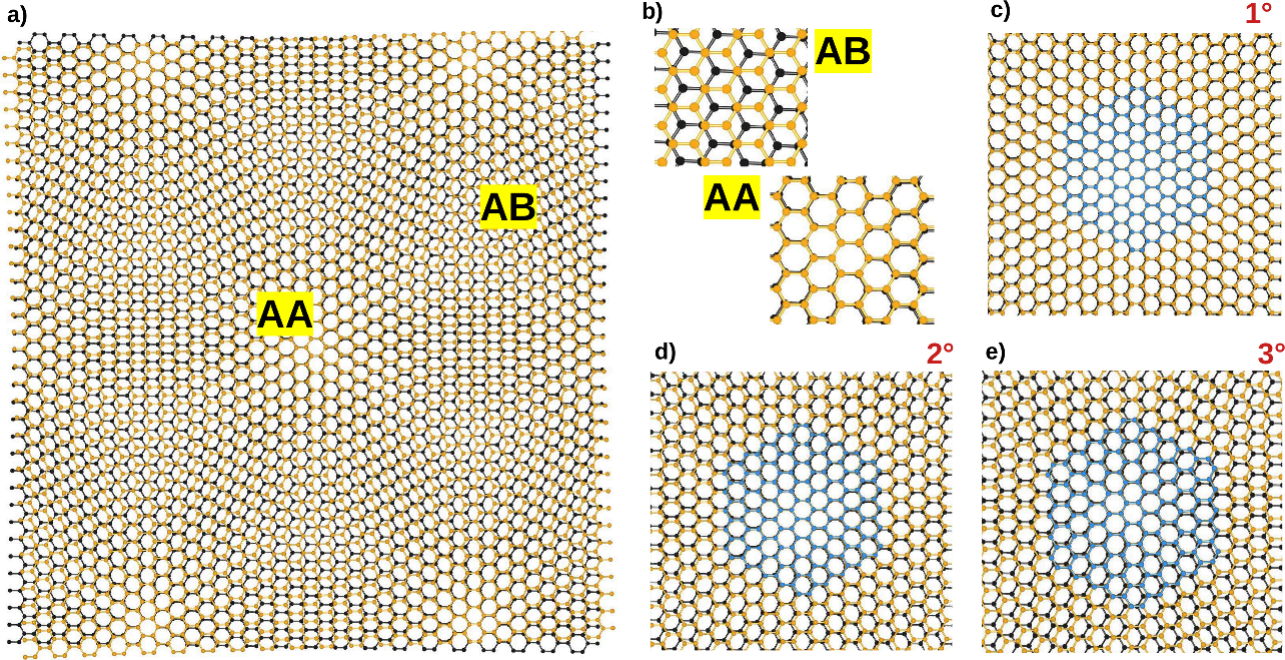
Top view of the graphene bilayer with the AA and AB regions (a, b) for several twisted angles (c–e). The blue regions (c–e) refer to the AA-stacked circumcircumcoronene (C_150_H_30_).

This work explores the magnetic moment in graphene bilayer nanoflakes, aiming to identify the conditions required for the emergence of magnetic dipole moments, thereby contributing to understanding why the magic angle in twisted bilayer graphene is approximately 1°. Bilayer graphene nanoflakes of three different sizes and rotation angles are studied, namely, C_192_H_48_, C_300_H_60_, and C_432_H_72_, as shown in Figure 2a–i and in Figure S1 of the Supporting Information (SI). Density Functional Theory along with the B3LYP^46^ functional, the def2-SVP basis set,^47^ and the D3BJ Grimme’s long-range correction^48^ were applied; i.e., the D3-B3LYP/def2-SVP level of theory was adopted. Note that this level of theory was recently employed in studies of planar nanoflakes, yielding accurate results^27,49^ in predicting ground spin state, geometrical parameters, and long-range interaction with adsorbed atoms. The Orca computational package^50,51^ was used in all calculations.

**Figure 2:**
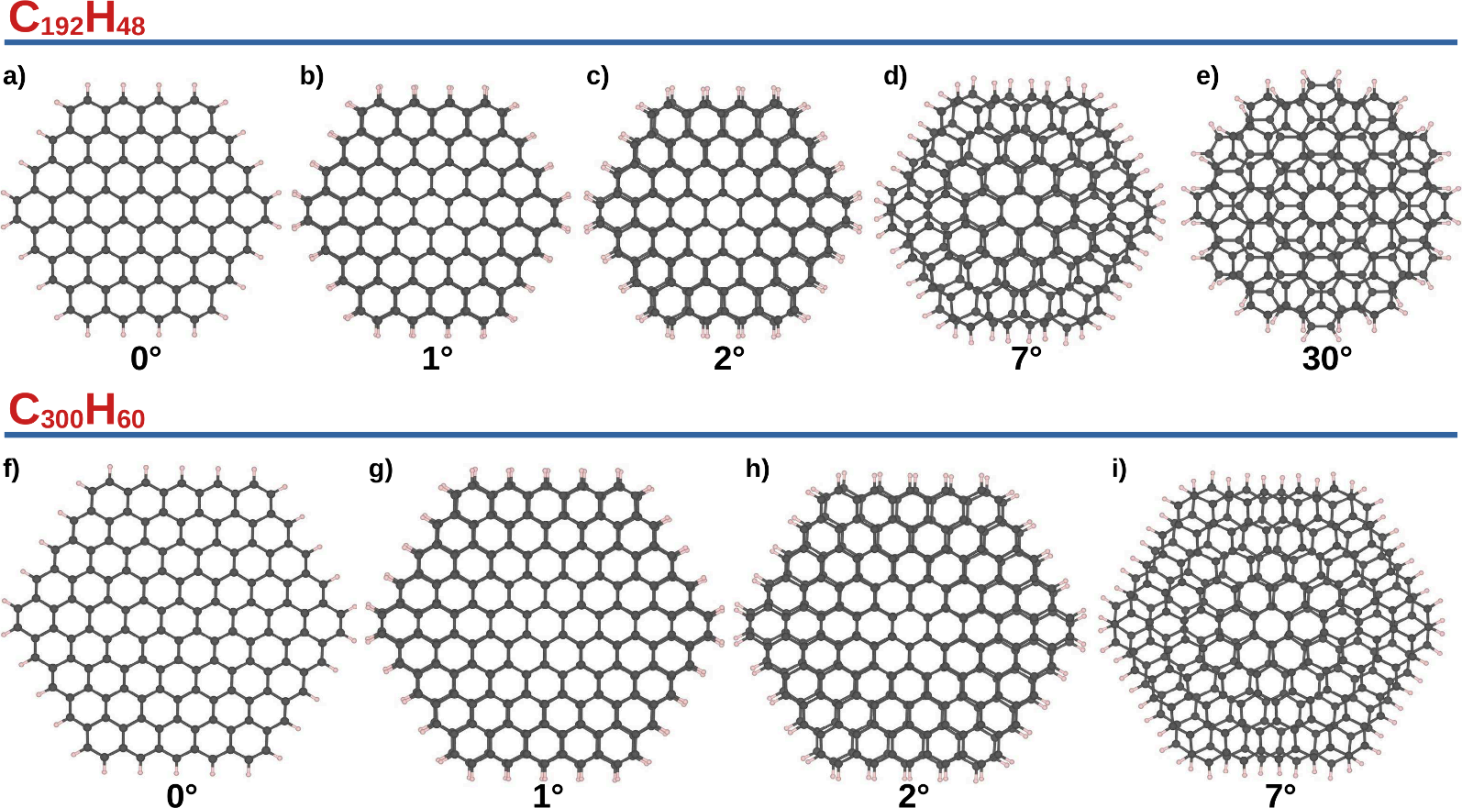
Top view of the graphene nanoflake bilayer for C_192_H_48_ (a–e) and C_300_H_60_ (f–i) structures at different twisted angles.

To determine the geometries of each graphene nanoflake layer, the isolated monolayers were first optimized individually. Subsequently, bilayer structures were constructed by stacking the optimized monolayers. The final configurations for the C_192_H_48_ and C_300_H_60_ systems, considering various twist angles, are presented in Figure 2, while the C_432_H_72_ structure with a 0° twist angle is included in Figure S1 of the SI.

To understand how the electronic structure changes, the Potential Energy Curves (PECs), considering the interlayer distance, were calculated for various angles (1°, 2°, 7°, and 30°) and different spin states (singlet and triplet). The PECs and the singlet–triplet total energy differences are gathered in Figures 3a,b and 4a,b, respectively, for C_192_H_48_ and C_300_H_60_ systems. Due to the size consistency of the level of theory employed here, the energy of the dissociated systems (the zero in the PEC graphics) is two times the one calculated by the isolated monolayer flake. Note that the twisted angle effect can be completely ignored for a large enough distance. One limitation of the level of theory used to build the PEC is the poor description of the interlayer interaction for distances far from equilibrium due to the single-reference conceptualization of the DFT. However, a good description of the region in the vicinity of the minimum in the PEC is expected.

**Figure 3:**
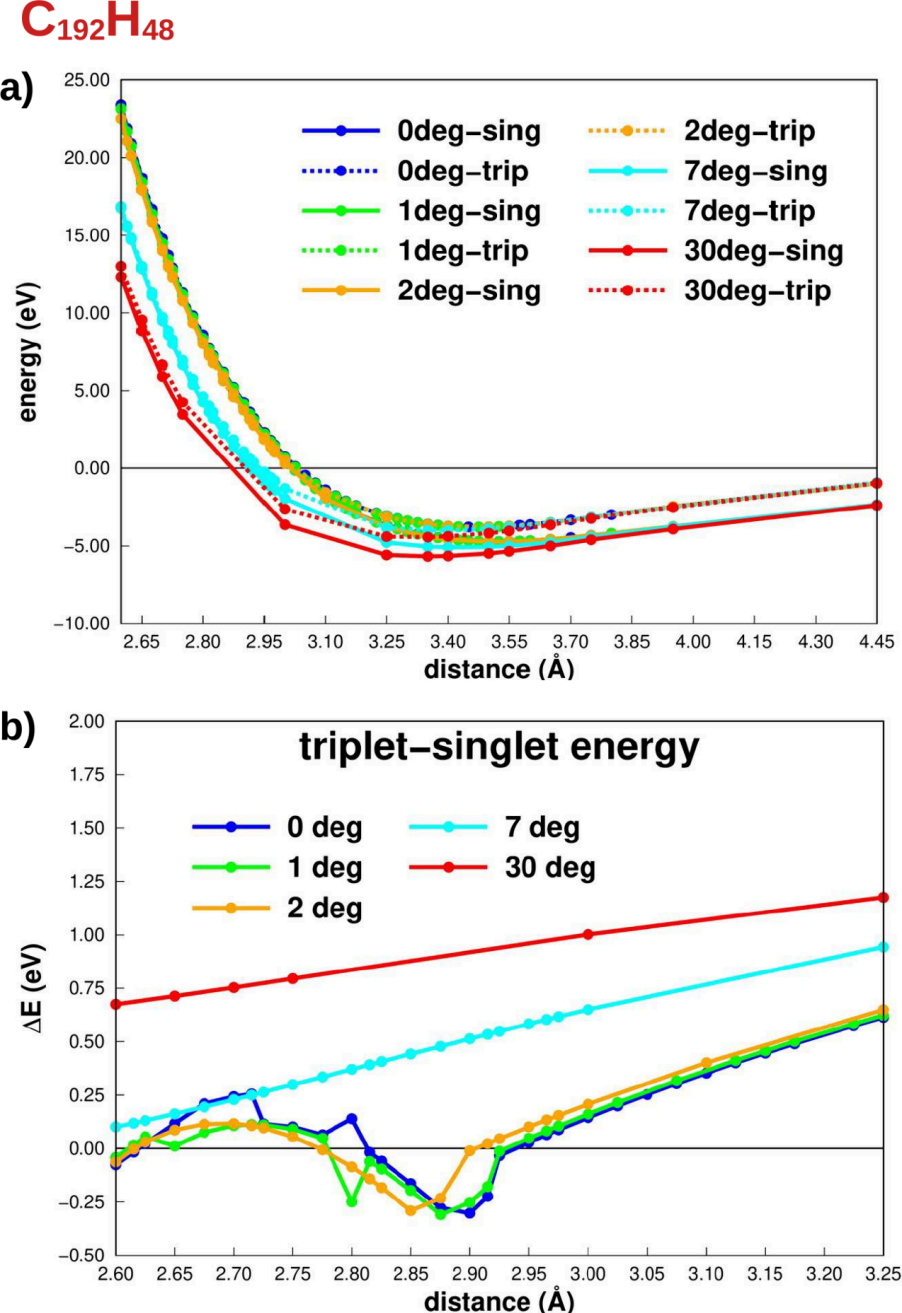
(a) Potential energy curves, considering the interlayer distance, of the C_192_H_48_ structure at different angles. (b) The singlet–triplet total energy difference, with negative values indicating a triplet ground state.

**Figure 4:**
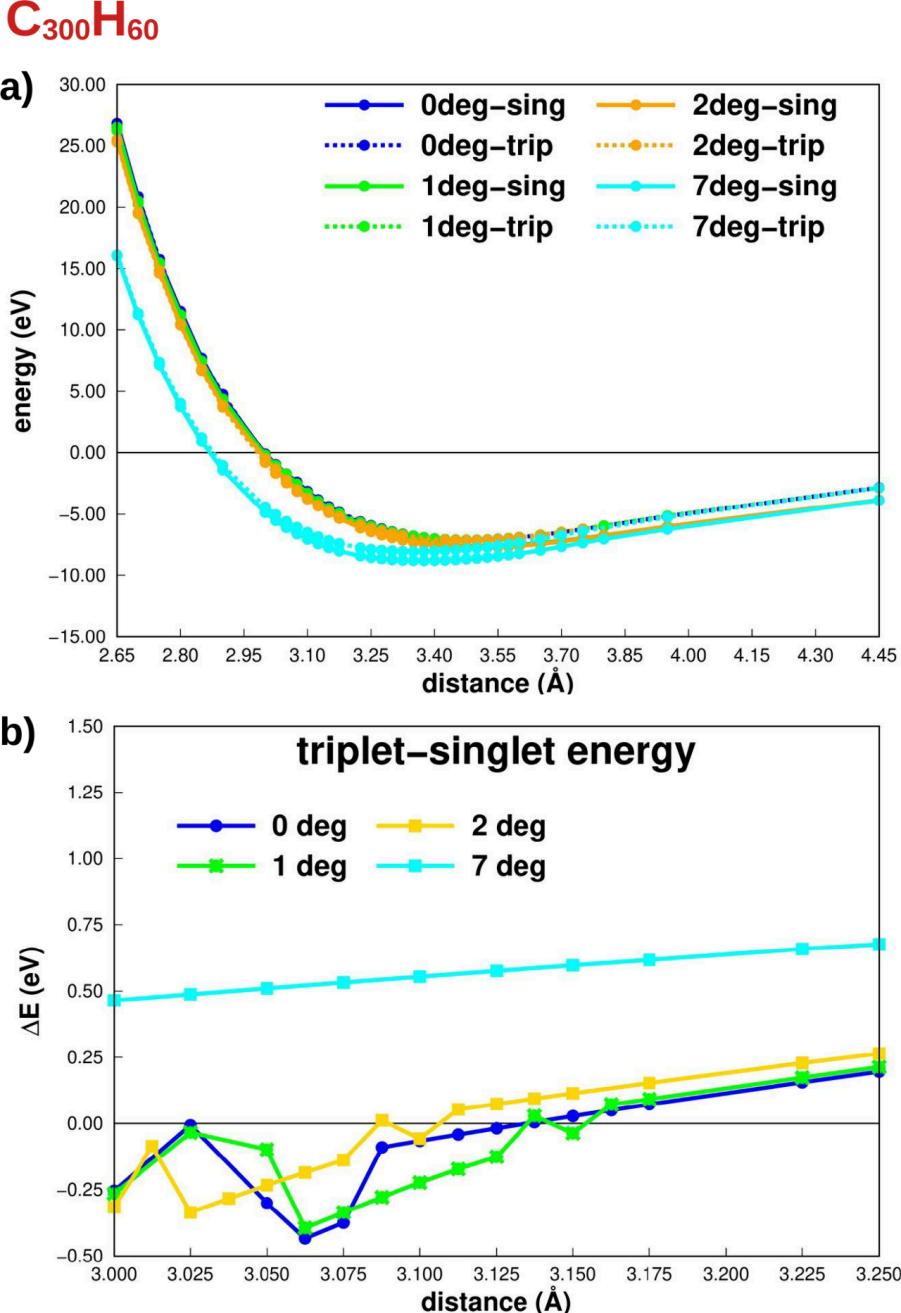
(a) Potential energy curves, considering the interlayer distance, of the C_300_H_60_ structure at different angles. (b) Singlet–triplet total energy difference whose negative values imply triplet as the ground state.

For all angles considered here, the ground spin state at the minimum of the PEC is singlet; i.e., no magnetic moment arises in the ground state geometry, as shown in panel (a) of Figures 3 and 4. These results agree with those obtained for the 7-triangulene bilayer at the equilibrium interlayer distance and AA stacking.^21^ Additionally, as the angle increases, the potential well becomes deeper; i.e., the structure is more stable for larger angles, for both singlet and triplet spin states. This result is expected, considering that part of the structure approaches the AB stacking for a larger twisted angle (see Figure 2d,e,i) and that the AB is more stable than the AA configuration.^52^ Consequently, the interlayer distance tends to decrease monotonically as the angle increases, approaching the AB stacking minimum interlayer distance (3.354 Å), as shown in Table 1 for the ground spin state (singlet).

**Table 1:** Interlayer Distance of the Ground State (Singlet) for C_192_H_48_ and C_300_H_60_ Systems with Different Twisted Angles

	interlayer distance^a^ (Å)	
angle	C_192_H_48_	C_300_H_60_
0°	3.520	3.516
1°	3.520	3.514
2°	3.519	3.508
7°	3.430	3.371
30°	3.346	3.357
AB	3.365	3.354

a Values obtained from a sixth-order polynomial adjusted to the distances close to the minimum of the PECs.

To better understand the behavior of the spin states outside the minimum region, the singlet–triplet total energy differences (Δ*E*) were plotted in Figures 3b and 4b. Negative values indicate that the ground spin state is a triplet one. For both cases, C_192_H_48_ and C_300_H_60_, the triplet spin-state energy is more negative than the singlet only in the repulsive region of the PEC (*d* < *d*_equi_) and for small angles (0°, 1°, and 2°). In other words, for the magnetic moment to arise, the interlayer distance must be less than the equilibrium distance. Notably, the maximum distance with a triplet as the ground state depends on the size and angle of the system. The distances between the layers are 2.932 and 3.132 Å for C_192_H_48_ and C_300_H_60_ with 0° and 2.900 and 3.100 Å with 2°, respectively. For both the C_192_H_48_ and C_300_H_60_ systems, the Δ*E* curve shows a minimum that occurs at different distances, which also depends on the system size and twist angle. For the C_300_H_60_ flake, the distance is 3.062 Å with 0° and 1°, while, with 2°, this interlayer distance is 3.0325 Å. For the C_192_H_48_ system, the distances are 2.900 Å with 0°, 2.887 Å with 1°, and 2.850 Å with 2°, respectively.

To confirm the trends discussed above, the singlet–triplet total energy differences were calculated for the C_432_H_72_ system, as detailed in the SI (Figure S2 of the SI). The largest interlayer distance with the triplet as the ground state was approximately 3.350 Å, which surpasses those obtained for the C_192_H_48_ and C_300_H_60_ systems and is close to the ground state distance for AB stacking (see Table 1). The minimum in the Δ*E* curve occurs at a distance of 3.232 Å, which is also larger compared to the C_192_H_48_ and C_300_H_60_ structures. Despite these larger distances, the triplet ground state still arises in the repulsive region of the PEC for all systems studied here relative to the AA ground state distance (Table 1). Thus, the repulsive interaction between flakes acts as a tuning parameter, changing the energy scenario and favoring a magnetic triplet ground state over the nonmagnetic configuration.

Although AA stacking is energetically less favorable than AB stacking, our calculations indicate that the structure remains in a local metastable state, as shown in Figures 3 and 4. Furthermore, the required out-of-plane pressure to maintain the necessary distance for the triplet spin-state in the C_300_H_60_ system at 1° is moderate, around −5.56 GPa, as discussed in the SI. Our results for nanoflakes agree with experiments on moiré superlattices of graphene encapsulated in h-BN,^53−55^ where it was shown that applying pressure increases the magic angle in the phase transition. This suggests that the configuration studied here could, in principle, be stabilized under experimentally feasible conditions.

In the DFT framework, the total energy decomposition can offer insights into the physical mechanisms driving spin polarization and magnetic moment formation. As illustrated in Figures S3 and S4 of the SI (for the C_300_H_60_ and C_432_H_72_ systems, respectively), the reduction (in magnitude) of the electronic kinetic energy and the increase (in magnitude) of the exchange-correlation energy near the minimum in Δ*E* serve as indicators of enhanced electronic correlation in the repulsive region, which triggers a Stoner instability when electrons interact, aligning with the mechanisms leading to spin polarization as discussed in ref (56). Notably, these changes are less pronounced in the triplet energy components, as also shown in Figures S3 and S4.

The energies of the highest occupied molecular orbital (HOMO) of the singlet, the semioccupied molecular orbitals (HOMO–1 and HOMO) for the triplet spin state, and the lowest molecular orbital (LUMO) are gathered in Figures S5 and S6 of the SI for the various interlayer distances for the C_192_H_48_ and C_300_H_60_ systems. Also shown in the SI (Figures S5 and S6) is the HOMO–LUMO gap for the same systems and distances. Notably, for the interlayer distance where the triplet to singlet spin-state transition occurs, the HOMO and the HOMO–1 of the triplet and the HOMO of the singlet assume the same values, with the same happening for the LUMO and HOMO–LUMO gap energies. Obviously, these limit distances coincide with those discussed in the previous paragraph, with small fluctuations, due to the difficulty of the DFT level in describing the LUMO energies.

The HOMO and LUMO behavior described above was also observed for carbon triangulene embedded in hexagonal boron nitride nanoflakes under applied electric and magnetic fields.^25^ At a specific external electric field value, where the triplet-to-quintet spin-state transition occurs, the orbital energies converge to the same values. Additionally, for a particular value of the applied electric field, the orbital occupation numbers of the singlet and triplet states tend to become similar, as observed in diatomic molecules.^57^ The conclusions presented here—that the singlet-to-triplet spin-state transition arises only in the repulsive region of the PEC—are consistent with spin-state transitions under an external electric field. In this context, the repulsion between the layers plays the role of the applied field, inducing the spin-state transition and, consequently, giving rise to the magnetic moment.

In addition to the confinement effect, the presence of a magnetic moment in the AA region in the twisted graphene could be explained by the smaller interlayer distance in the AB region compared with the AA region. In other words, the AA regions might be under strain due to the AB configuration, and this repulsive effect could give rise to a magnetic moment. However, based on the findings presented here, this potential explanation contrasts with the results from Molecular Dynamics (MD) simulations^58,59^ of fully relaxed bilayer graphene, which shows that, on average, the interlayer distance in the AA regions is practically the same as that in bilayer AA-stacked graphene (0°). However, from an experimental perspective, the graphene monolayer is typically deposited on a substrate,^45^ which could limit the mobility of the free-standing bilayer used in previous MD simulations.^58,59^

Figure 5a–f shows the spin density for the triplet spin state in the C_192_H_48_ and C_300_H_60_ systems (see Figure S7 of the SI for a side view). The interlayer distances correspond to those yielded by the minimum Δ*E* value in Figures 3b and 4b. Notably, for all small twisted angles (0°–2°), the spin density exhibits a similar distribution throughout the systems. The spin-up density is predominant in the nanoflakes, presenting a nonsymmetrical distribution that breaks the antiferromagnetic (ferrimagnetic) coupling between sublattices A and B of the same layer, which is expected for closed-shell (open-shell) graphene nanoflakes.^25−27,60^ This behavior is similar to that observed for graphene nanoflakes with AA and AB stacking under in-plane uniaxial strain,^61^ where the strain modifies the magnetic moments providing a complex ordering that has a mix of ferromagnetic and antiferromagnetic couplings between the two layers. The flake’s edge plays an important role, where the most intense spin density is most concentrated in this region. In periodic twisted bilayer graphene, this confinement effect is produced by the interface between the AA and AB stacking, enabling ferromagnetic coupling between different AA regions.

**Figure 5:**
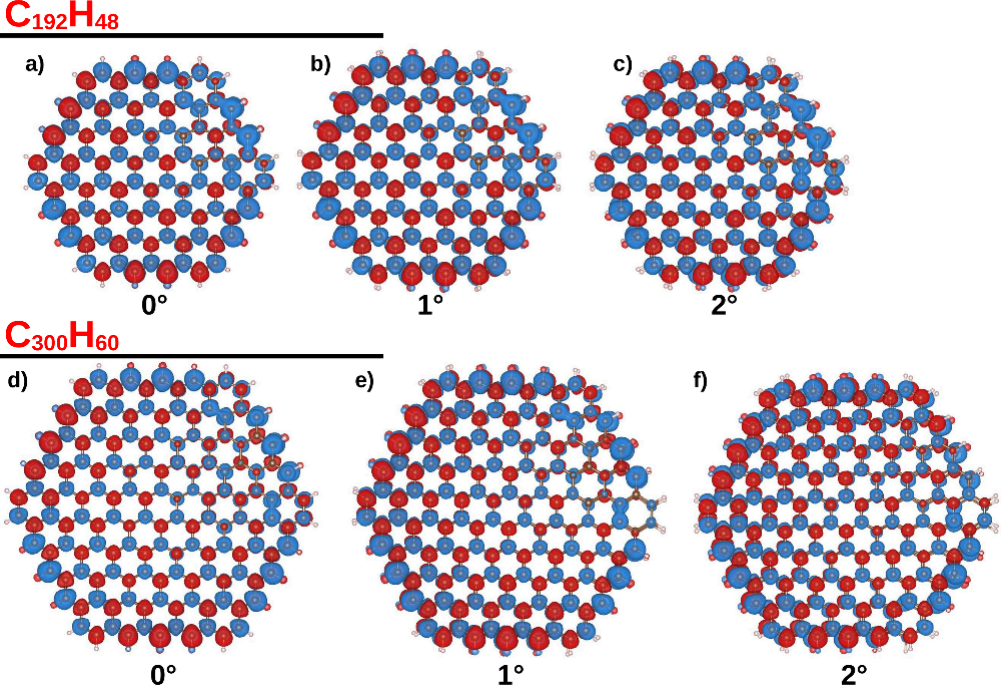
Spin density of the C_192_H_48_ (a–c) and C_300_H_60_ (d–f) systems at triplet spin state. The blue and red colors refer to the up and down spin, respectively. The interlayer distances were those that produced the lowest Δ*E* value in Figures 3b and 4b.

In summary, our results indicate that structural and electronic conditions govern the magnetic properties of twisted graphene bilayers. The repulsive interaction between the layers in the AA region proves to be a fundamental ingredient in the emergence of the magnetic moment from the singlet–triplet spin state transition. Furthermore, the size of the AA stacking region is directly connected with the distance at which the spin state transition occurs; i.e., a smaller AA region implies a shorter interlayer distance. Therefore, selecting the appropriate twist angle is crucial for generating a sufficiently large AA stacking region, allowing the spin state transition to occur at a distance similar to that induced by the AB stacking. Thus, based on our findings, the twisted angle close to 1° fulfills the necessary conditions for the emergence of the magnetic moment. Spin density analysis confirms that edge effects play a significant role, indicating that confinement introduced by the AB region is essential to the emergence of the magnetic moment. Clearly, the border effect provided by the flakes is of a different nature compared to that of periodic twisted graphene and is primarily responsible for the properties arising from the edge states. Nevertheless, the conclusions presented here suggest that the magnetic properties of these systems, particularly the transition of the magnetic moment, can be finely tuned through the application of strain. The effect of out-of-plane strain on the system would be similar to that observed in the repulsive region of the PEC, where increased electronic interactions play a key role in influencing the magnetic behavior. This highlights a promising pathway for controlling singlet–triplet splitting and tailoring the magnetism of bilayer nanoflakes for potential applications in nanoscale devices.

## Supporting Information Available

Hydrostatic pressure calculation from harmonic approximation (Section 1); top view of the C_432_H_72_ graphene nanoflake with 0° of twisted angle (Figure S1); singlet and triplet total energy difference versus interlayer distances for the C_432_H_72_ graphene nanoflake (Figure S2); DFT energy partition versus the interlayer distance for C_300_H_60_ and C_432_H_72_ systems (Figures S3 and S4); orbital energies (HOMO–1, HOMO, and LUMO) and HOMO–LUMO energy gap for C_192_H_48_ and C_300_H_60_ graphene nanoflakes (Figures S5 and S6); side view of the spin density of the C_300_H_60_ nanoflake with 0°, 1°, and 2° twisted angles (Figure S7) (PDF)
Transparent Peer Review report available (PDF)

## References

[ref1] Burkard G.; Engel H.-A.; Loss D. Spintronics and quantum dots for quantum computing and quantum communication. Fortschr. Phys. 2000, 48, 965–986. 10.1002/1521-3978(200009)48:9/11<965::AID-PROP965>3.0.CO;2-V.

[ref2] Borissov A.; Maurya Y. K.; Moshniaha L.; Wong W.-S.; Zyła-Karwowska M.; Stepien M. Recent advances in heterocyclic nanographenes and other polycyclic heteroaromatic compounds. Chem. Rev. 2022, 122, 565–788. 10.1021/acs.chemrev.1c00449. 34850633 PMC8759089

[ref3] Ruffieux P.; Wang S.; Yang B.; Sánchez-Sánchez C.; Liu J.; Dienel T.; Talirz L.; Shinde P.; Pignedoli C. A.; Passerone D.; et al. On-surface synthesis of graphene nanoribbons with zigzag edge topology. Nature 2016, 531, 489–492. 10.1038/nature17151. 27008967

[ref4] Mishra S.; Beyer D.; Eimre K.; Liu J.; Berger R.; Gröning O.; Pignedoli C. A.; Müllen K.; Fasel R.; Feng X.; et al. Synthesis and characterization of π-extended triangulene. J. Am. Chem. Soc. 2019, 141, 10621–10625. 10.1021/jacs.9b05319. 31241927

[ref5] Pavliček N.; Mistry A.; Majzik Z.; Moll N.; Meyer G.; Fox D. J.; Gross L. Synthesis and characterization of triangulene. Nature Nanotechnol. 2017, 12, 308–311. 10.1038/nnano.2016.305. 28192389

[ref6] Song S.; Su J.; Telychko M.; Li J.; Li G.; Li Y.; Su C.; Wu J.; Lu J. On-surface synthesis of graphene nanostructures with π-magnetism. Chem. Soc. Rev. 2021, 50, 3238–3262. 10.1039/D0CS01060J. 33481981

[ref7] Novoselov K. S.; Geim A. K.; Morozov S. V.; Jiang D.-e.; Zhang Y.; Dubonos S. V.; Grigorieva I. V.; Firsov A. A. Electric field effect in atomically thin carbon films. Science 2004, 306, 666–669. 10.1126/science.1102896. 15499015

[ref8] Geim A. K.; Novoselov K. S. The rise of graphene. Nat. Mater. 2007, 6, 183–191. 10.1038/nmat1849. 17330084

[ref9] Ullah S.; Shi Q.; Zhou J.; Yang X.; Ta H. Q.; Hasan M.; Ahmad N. M.; Fu L.; Bachmatiuk A.; Rümmeli M. H. Advances and trends in chemically doped graphene. Adv. Mater. Interfaces. 2020, 7, 2000999 10.1002/admi.202000999.

[ref10] Paz W. S.; Scopel W. L.; Freitas J. C. On the connection between structural distortion and magnetism in graphene with a single vacancy. Solid State Commun. 2013, 175–176, 71–75. 10.1016/j.ssc.2013.05.004.

[ref11] Li R.; Zhang M.; Fu X.; Gao J.; Huang C.; Li Y. Research of low-dimensional carbon-based magnetic materials. ACS Appl. Electron. Mater. 2022, 4, 3263–3277. 10.1021/acsaelm.2c00407.

[ref12] Li C.; Sun X.; Yuan P.; Wang F.; Niu C.; Cui B.; Jia Y. Design of atomically localized magnetic moment by adatoms chemisorbed on graphene. Phys. Lett. A 2024, 504, 129435 10.1016/j.physleta.2024.129435.

[ref13] Guan Y.; Dutreix C.; González-Herrero H.; Ugeda M. M.; Brihuega I.; Katsnelson M. I.; Yazyev O. V.; Renard V. T. Observation of Kekulé vortices around hydrogen adatoms in graphene. Nat. Commun. 2024, 15, 2927 10.1038/s41467-024-47267-8. 38575594 PMC10995122

[ref14] González-Herrero H.; Gómez-Rodríguez J. M.; Mallet P.; Moaied M.; Palacios J. J.; Salgado C.; Ugeda M. M.; Veuillen J.-Y.; Yndurain F.; Brihuega I. Atomic-scale control of graphene magnetism by using hydrogen atoms. Science 2016, 352, 437–441. 10.1126/science.aad8038. 27102478

[ref15] Lieb E. H. Two Theorems on the Hubbard Model. Phys. Rev. Lett. 1989, 62, 1201 10.1103/PhysRevLett.62.1201. 10039602

[ref16] Hubbard J. Electron correlations in narrow energy bands. Proc. R. Soc. London A. Math. Phys. Sci. 1963, 276, 238–257. 10.1098/rspa.1963.0204.

[ref17] Calupitan J. P.; Berdonces-Layunta A.; Aguilar-Galindo F.; Vilas-Varela M.; Peña D.; Casanova D.; Corso M.; de Oteyza D. G.; Wang T. Emergence of π-Magnetism in Fused Aza-Triangulenes: Symmetry and Charge Transfer Effects. Nano Lett. 2023, 23, 9832–9840. 10.1021/acs.nanolett.3c02586. 37870305 PMC10722538

[ref18] Pavliček N.; Mistry A.; Majzik Z.; Moll N.; Meyer G.; Fox D. J.; Gross L. Synthesis and characterization of triangulene. Nat. Nanotechnol. 2017, 12, 308–311. 10.1038/nnano.2016.305. 28192389

[ref19] Mishra S.; Beyer D.; Eimre K.; Liu J.; Berger R.; Gröning O.; Pignedoli C. A.; Müllen K.; Fasel R.; Feng X.; et al. Synthesis and Characterization of π-Extended Triangulene. J. Am. Chem. Soc. 2019, 141, 10621–10625. 10.1021/jacs.9b05319. 31241927

[ref20] Su J.; Telychko M.; Hu P.; Macam G.; Mutombo P.; Zhang H.; Bao Y.; Cheng F.; Huang Z.-Q.; Qiu Z.; et al. Atomically precise bottom-up synthesis of π-extended [5]triangulene. Sci. Adv. 2019, 5, eaav7717 10.1126/sciadv.aav7717. 31360763 PMC6660211

[ref21] Sharifian M.; Hoseini S.; Faizabadi E. Ground state magnetic properties in AA-stacking bilayer graphene quantum dots using Lieb’s theorem. J. Magn. Magn. Mater. 2019, 477, 427–433. 10.1016/j.jmmm.2019.01.057.

[ref22] Mishra S.; Xu K.; Eimre K.; Komber H.; Ma J.; Pignedoli C. A.; Fasel R.; Feng X.; Ruffieux P. Synthesis and characterization of [7]triangulene. Nanoscale 2021, 13, 1624–1628. 10.1039/D0NR08181G. 33443270

[ref23] Song S.; Su J.; Telychko M.; Li J.; Li G.; Li Y.; Su C.; Wu J.; Lu J. On-surface synthesis of graphene nanostructures with π-magnetism. Chem. Soc. Rev. 2021, 50, 3238–3262. 10.1039/D0CS01060J. 33481981

[ref24] Chabi S.; Guler Z.; Brearley A. J.; Benavidez A. D.; Luk T. S. The Creation of True Two-Dimensional Silicon Carbide. Nanomaterials 2021, 11, 1799 10.3390/nano11071799. 34361184 PMC8308388

[ref25] Filho L. F.; Terroso C.; de Souza F.; Paz W.; Pansini F. Spin state engineering of triangulene graphene embedded in h-BN nanoflake. Carbon 2023, 213, 118186 10.1016/j.carbon.2023.118186.

[ref26] de Souza F.; Pansini F.; Ambrozio A. R.; Freitas J.; Scopel W. L. others NMR spectral parameters of open-and closed-shell graphene nanoflakes: Orbital and hyperfine contributions. Carbon 2022, 191, 374–383. 10.1016/j.carbon.2022.01.045.

[ref27] Filho L. F.; Morais W. P.; Batista N. N.; de Souza F. A. L.; Varandas A. J. C.; Paz W. S.; Pansini F. N. N. Hydrogen-designed spin-states of 2D silicon carbide and graphene nanostructures. Phys. Chem. Chem. Phys. 2024, 26, 26576–26584. 10.1039/D4CP02762K. 39400278

[ref28] Zhao C.; Catarina G.; Zhang J.-J.; Henriques J. C.; Yang L.; Ma J.; Feng X.; Gröning O.; Ruffieux P.; Fernández-Rossier J.; et al. Tunable topological phases in nanographene-based spin-1/2 alternating-exchange Heisenberg chains. Nat. Nanotechnol. 2024, 19, 1789–1795. 10.1038/s41565-024-01805-z. 39468357

[ref29] Fang T.; Zhang T.; Hu T.; Wang Z. Atomic-Limit Mott Insulator in [4] Triangulene Frameworks. Nano Lett. 2024, 24, 3059–3066. 10.1021/acs.nanolett.3c04675. 38426713

[ref30] Li L.; Qin R.; Li H.; Yu L.; Liu Q.; Luo G.; Gao Z.; Lu J. Functionalized graphene for high-performance two-dimensional spintronics devices. ACS Nano 2011, 5, 2601–2610. 10.1021/nn102492g. 21395280

[ref31] Yazyev O. V.; Helm L. Defect-induced magnetism in graphene. Physical Review B—Condensed Matter and Materials Physics 2007, 75, 125408 10.1103/PhysRevB.75.125408.

[ref32] Tada K.; Haruyama J.; Yang H.; Chshiev M.; Matsui T.; Fukuyama H. Ferromagnetism in hydrogenated graphene nanopore arrays. Physical review letters 2011, 107, 217203 10.1103/PhysRevLett.107.217203. 22181918

[ref33] Feng Q.; Zheng Y.; Li J.; Jiang L.; Lin Y.; Ye Q.; Chen L.; Huang Z. Observation of ferromagnetic ordering by fragmenting fluorine clusters in highly fluorinated graphene. Carbon 2018, 132, 691–697. 10.1016/j.carbon.2018.02.097.

[ref34] Cao Y.; Fatemi V.; Demir A.; Fang S.; Tomarken S. L.; Luo J. Y.; Sanchez-Yamagishi J. D.; Watanabe K.; Taniguchi T.; Kaxiras E.; et al. Correlated insulator behaviour at half-filling in magic-angle graphene superlattices. Nature 2018, 556, 80–84. 10.1038/nature26154. 29512654

[ref35] Cao Y.; Fatemi V.; Fang S.; Watanabe K.; Taniguchi T.; Kaxiras E.; Jarillo-Herrero P. Unconventional superconductivity in magic-angle graphene superlattices. Nature 2018, 556, 43–50. 10.1038/nature26160. 29512651

[ref36] Po H. C.; Zou L.; Vishwanath A.; Senthil T. Origin of Mott insulating behavior and superconductivity in twisted bilayer graphene. Phys. Rev. X 2018, 8, 031089 10.1103/PhysRevX.8.031089.

[ref37] Yankowitz M.; Chen S.; Polshyn H.; Zhang Y.; Watanabe K.; Taniguchi T.; Graf D.; Young A. F.; Dean C. R. Tuning superconductivity in twisted bilayer graphene. Science 2019, 363, 1059–1064. 10.1126/science.aav1910. 30679385

[ref38] Lu X.; Stepanov P.; Yang W.; Xie M.; Aamir M. A.; Das I.; Urgell C.; Watanabe K.; Taniguchi T.; Zhang G.; et al. Superconductors, orbital magnets and correlated states in magic-angle bilayer graphene. Nature 2019, 574, 653–657. 10.1038/s41586-019-1695-0. 31666722

[ref39] Sharpe A. L.; Fox E. J.; Barnard A. W.; Finney J.; Watanabe K.; Taniguchi T.; Kastner M.; Goldhaber-Gordon D. Emergent ferromagnetism near three-quarters filling in twisted bilayer graphene. Science 2019, 365, 605–608. 10.1126/science.aaw3780. 31346139

[ref40] Pixley J. H.; Andrei E. Y. Ferromagnetism in magic-angle graphene. Science 2019, 365, 543–543. 10.1126/science.aay3409.

[ref41] Polshyn H.; Yankowitz M.; Chen S.; Zhang Y.; Watanabe K.; Taniguchi T.; Dean C. R.; Young A. F. Large linear-in-temperature resistivity in twisted bilayer graphene. Nat. Phys. 2019, 15, 1011–1016. 10.1038/s41567-019-0596-3.

[ref42] Serlin M.; Tschirhart C.; Polshyn H.; Zhang Y.; Zhu J.; Watanabe K.; Taniguchi T.; Balents L.; Young A. Intrinsic quantized anomalous Hall effect in a moiré heterostructure. Science 2020, 367, 900–903. 10.1126/science.aay5533. 31857492

[ref43] Lisi S.; Lu X.; Benschop T.; de Jong T. A.; Stepanov P.; Duran J. R.; Margot F.; Cucchi I.; Cappelli E.; Hunter A.; et al. Observation of flat bands in twisted bilayer graphene. Nat. Phys. 2021, 17, 189–193. 10.1038/s41567-020-01041-x.

[ref44] Rakkesh R. A.; Rebecca P. B.; Naveen T.; Durgalakshmi D.; Balakumar S. Twisted Bilayer Graphene: A Journey Through Recent Advances and Future Perspectives. Part. Syst. Charact. 2024, 41, 2300125 10.1002/ppsc.202300125.

[ref45] Saumya K.; Naskar S.; Mukhopadhyay T. Magic’of twisted multi-layered graphene and 2D nano-heterostructures. Nano Fut 2023, 7, 032005 10.1088/2399-1984/acf0a9.

[ref46] Becke A. D. Density-functional thermochemistry. III. The role of exact exchange. J. Chem. Phys. 1993, 98, 5648–5652. 10.1063/1.464913.

[ref47] Weigend F.; Ahlrichs R. Balanced basis sets of split valence, triple zeta valence and quadruple zeta valence quality for H to Rn: Design and assessment of accuracy. Phys. Chem. Chem. Phys. 2005, 7, 3297 10.1039/b508541a. 16240044

[ref48] Grimme S.; Ehrlich S.; Goerigk L. Effect of the damping function in dispersion corrected density functional theory. J. Comput. Chem. 2011, 32, 1456–1465. 10.1002/jcc.21759. 21370243

[ref49] Sergio C.; Pansini F.; de Campos M. The relationship between hydrogen storage capacity and 4d transition metal-carbon surface binding energy. Chem. Phys. Lett. 2024, 846, 141338 10.1016/j.cplett.2024.141338.

[ref50] Neese F. The ORCA program system. Wiley Interdiscip. Rev. Comput. Mol. Sci. 2012, 2, 73–78. 10.1002/wcms.81.

[ref51] Neese F. Software update: the ORCA program system, version 4.0. Wiley Interdiscip. Rev. Comput. Mol. Sci. 2018, 8, e1327 10.1002/wcms.1327.

[ref52] Rakhmanov A.; Rozhkov A.; Sboychakov A.; Nori F. Instabilities of the AA-stacked graphene bilayer. Phys. Rev. Lett. 2012, 109, 206801 10.1103/PhysRevLett.109.206801. 23215515

[ref53] Yankowitz M.; Jung J.; Laksono E.; Leconte N.; Chittari B. L.; Watanabe K.; Taniguchi T.; Adam S.; Graf D.; Dean C. R. Dynamic band-structure tuning of graphene moiré superlattices with pressure. Nature 2018, 557, 404–408. 10.1038/s41586-018-0107-1. 29769674

[ref54] Yankowitz M.; Chen S.; Polshyn H.; Zhang Y.; Watanabe K.; Taniguchi T.; Graf D.; Young A. F.; Dean C. R. Tuning superconductivity in twisted bilayer graphene. Science 2019, 363, 1059–1064. 10.1126/science.aav1910. 30679385

[ref55] Hou Y.; Zhou J.; Xue M.; Yu M.; Han Y.; Zhang Z.; Lu Y. Strain Engineering of Twisted Bilayer Graphene. Rise of Strain-Twistronics. Small 2024, 2311185 10.1002/smll.202311185. PMC12272032 38616775

[ref56] Gonzalez-Arraga L. A.; Lado J.; Guinea F.; San-Jose P. Electrically controllable magnetism in twisted bilayer graphene. Physical review letters 2017, 119, 107201 10.1103/PhysRevLett.119.107201. 28949176

[ref57] Pansini F. N. N.; de Souza F. A. L.; Campos C. T. Molecules under external electric field: On the changes in the electronic structure and validity limits of the theoretical predictions. J. Comput. Chem. 2018, 39, 1561–1567. 10.1002/jcc.25229. 29676469

[ref58] Qiu W.; Zhang B.; Sun Y.; He L.; Ni Y. Atomic reconstruction enabled coupling between interlayer distance and twist in van der Waals bilayers. Extreme Mech. Lett. 2024, 69, 102159 10.1016/j.eml.2024.102159.

[ref59] Dai S.; Xiang Y.; Srolovitz D. J. Twisted bilayer graphene: Moiré with a twist. Nano Lett. 2016, 16, 5923–5927. 10.1021/acs.nanolett.6b02870. 27533089

[ref60] Fernández-Rossier J.; Palacios J. J. Magnetism in graphene nanoislands. Phys. Rev. Lett. 2007, 99, 177204 10.1103/PhysRevLett.99.177204. 17995364

[ref61] Nascimento J. S.; da Costa D. R.; Zarenia M.; Chaves A.; Pereira J. M. Magnetic properties of bilayer graphene quantum dots in the presence of uniaxial strain. Phys. Rev. B 2017, 96, 115428 10.1103/PhysRevB.96.115428.

