# Supplementary material for: Magnetic Moment and Spin-State Transitions in Twisted Graphene Nanostructures
Source: J Phys Chem Lett. 2025 Feb 18;16(8):1994–2000. doi: 10.1021/acs.jpclett.4c03542 (PMC11873933; doi:10.1021/acs.jpclett.4c03542)
Supplement: Supplementary file 1 — jz4c03542_si_001.pdf [file jz4c03542_si_001.pdf]

Supplementary Materials for: Magnetic Moment and Spin-State Transitions in Twisted Graphene Nanostructures

F. N. N. Pansini,*,† F. A. L. de Souza,‡ Wendel S. Paz,† and V. C. Mota†

†*Departamento de Física, Universidade Federal do Espírito Santo, Vitória, 29075-910, Brazil*

‡ *Instituto Federal de Educação, Ciência e Tecnologia do Espírito Santo, Ibatiba, 29395-000, Brazil*

E-mail: fernando.pansini@ufes.br

1 Out-of-plane Pressure

A quadratic behavior of the total energy (in eV) profile as a function of strain (ε) for the $C_{300}H_{60}$ system is presented in Figure S1, considering the interlayer distance close to the minimum. Thus, the energy profile fitted to a quadratic equation is:

$$E(\varepsilon) = 202.230\varepsilon^2 - 1.233\varepsilon - 311767.8,$$

where $\varepsilon = \frac{(h-h_0)}{h_0}$ and $h_0 = 3.516$ is the equilibrium interlayer distance. With the above equation, the Young's modulus under linear strain conditions [1] can be calculated:

$$Y = \frac{1}{V_0}\frac{\partial^2 E}{\partial \varepsilon^2}.$$

Here, $V_0 = A_0 \times h_0$ represents the equilibrium volume of the $C_{300}H_{60}$ system, where the area of the hexagonal nanoflake (A_0) remains unchanged. The equilibrium area is calculated as:

$$A_0 = \frac{3\sqrt{3}}{2}d^2,$$

where $d = 12.29$ Å is the side length of the hexagonal flake. Thus, the calculated Young's modulus is $Y = 46.97$ GPa. It is important to note that the harmonic approximation employed here is valid only for small variations in the interlayer distance, which was assumed in our model.

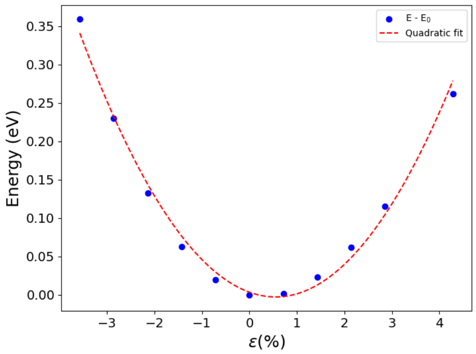

The relative energy (E = E_{strain} - E_0) vs. distance between the layers (h); E_0 represents the energy at the equilibrium spacing between the layers.

The strain-induced out-of-plane pressure, P, resulting from variations in h, is given by:

$$P = Y \times \left(\frac{h-h_0}{h_0}\right).$$

For the case of $C_{300}H_{60}$, using $d = 12.29$ Å and $h = 3.100$ Å (see text for details), the calculated pressure is $P = -5.56$ GPa. It is important to highlight that these values are consistent with those applied in 2D bilayer graphene systems [2-4].

1) Ghosh, P., Kahaly, M. U., & Waghmare, U. V. Atomic and electronic structures, elastic properties, and optical conductivity of bulk Te and Te nanowires: A first-principles study. Physical Review B 2007, 75(24), 245437. (2) Carr, S.; Fang, S.; Jarillo-Herrero, P.; Kaxiras, E. Pressure dependence of the magic twist angle in graphene superlattices. Physical Review B 2018, 98, 085144. (3) Szentpéteri, B.; Rickhaus, P.; de Vries, F. K.; Márffy, A.; Fulop, B.; Tóvári, E.; Watanabe, K.; Taniguchi, T.; Kormányos, A.; Csonka, S. et al. Tailoring the band structure of twisted double bilayer graphene with pressure. Nano Letters 2021, 21, 8777–8784. (4) Yuan Hou, Jingzhuo Zhou, Minmin Xue, Maolin Yu, Ying Han, Zhuhua Zhang, and Yang Lu, Strain Engineering of Twisted Bilayer Graphene: The Rise of Strain-Twistronics. Small, 2024, 2311185

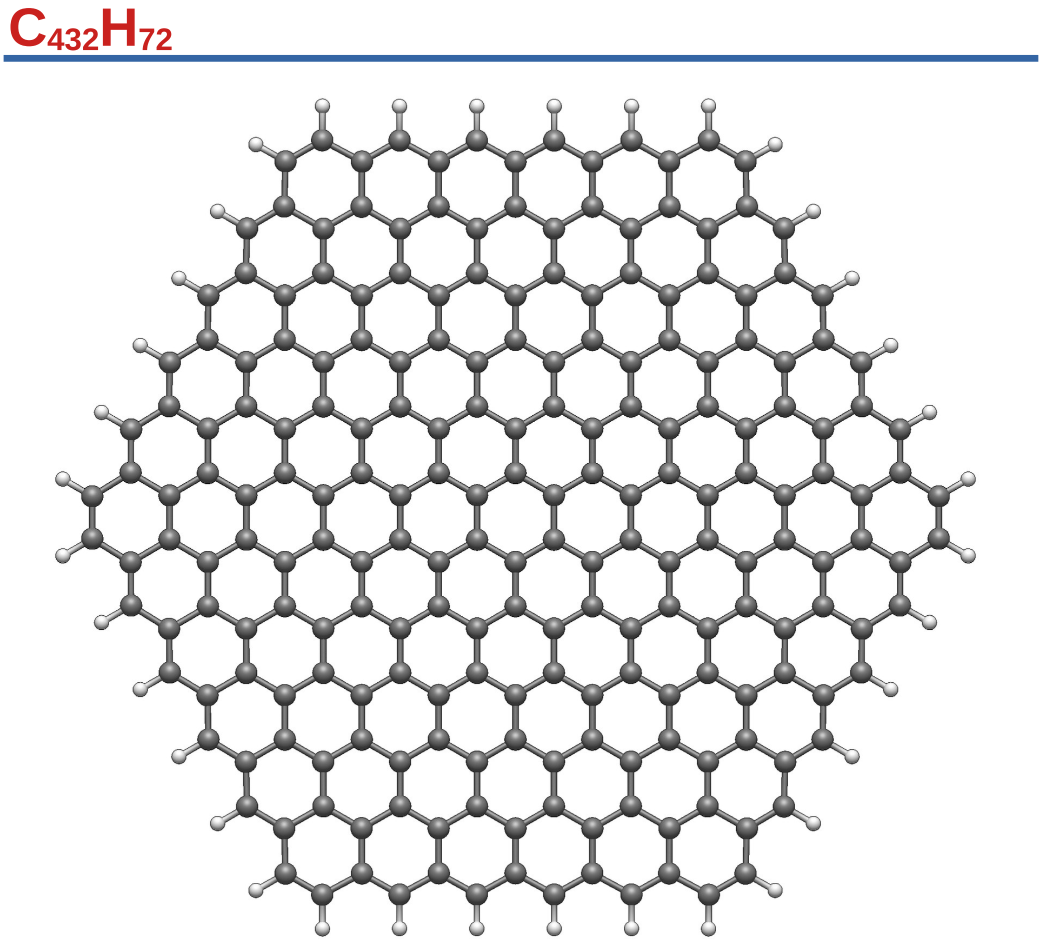

Figure S1: Top view of the $C_{432}H_{72}$ graphene nanoflake bilayer with 0° of twisted angle.

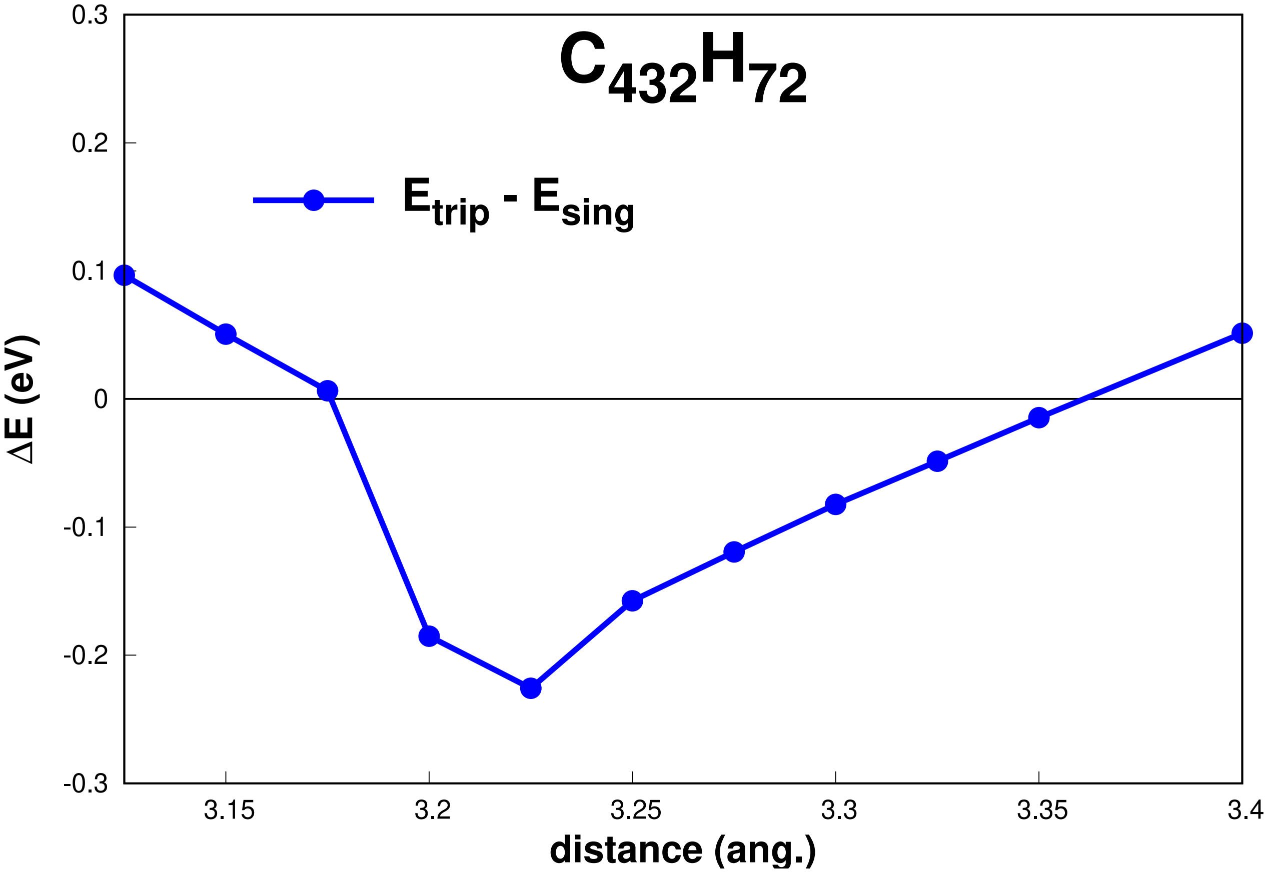

Figure S2: Singlet and triplet total energy difference versus interlayer distances for the $C_{432}H_{72}$ graphene nanoflake.

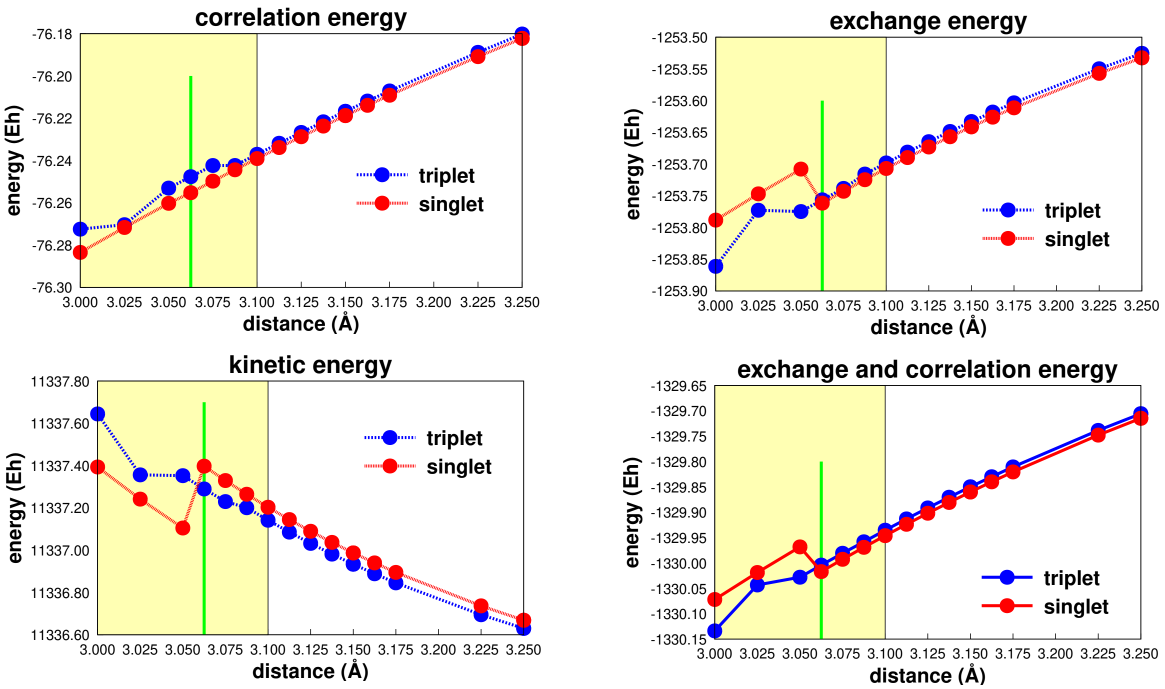

Figure S3: DFT energy partition versus the interlayer distance for $C_{300}H_{60}$ systems.

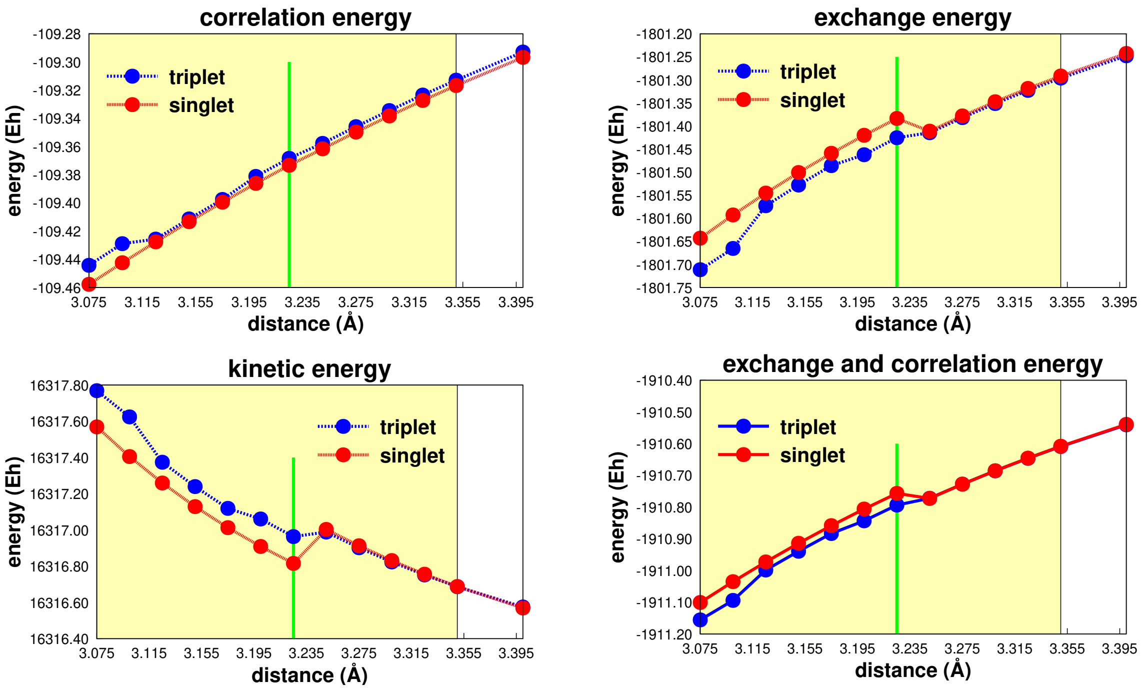

Figure S4: DFT energy partition versus the interlayer distance for $C_{432}H_{72}$ systems.

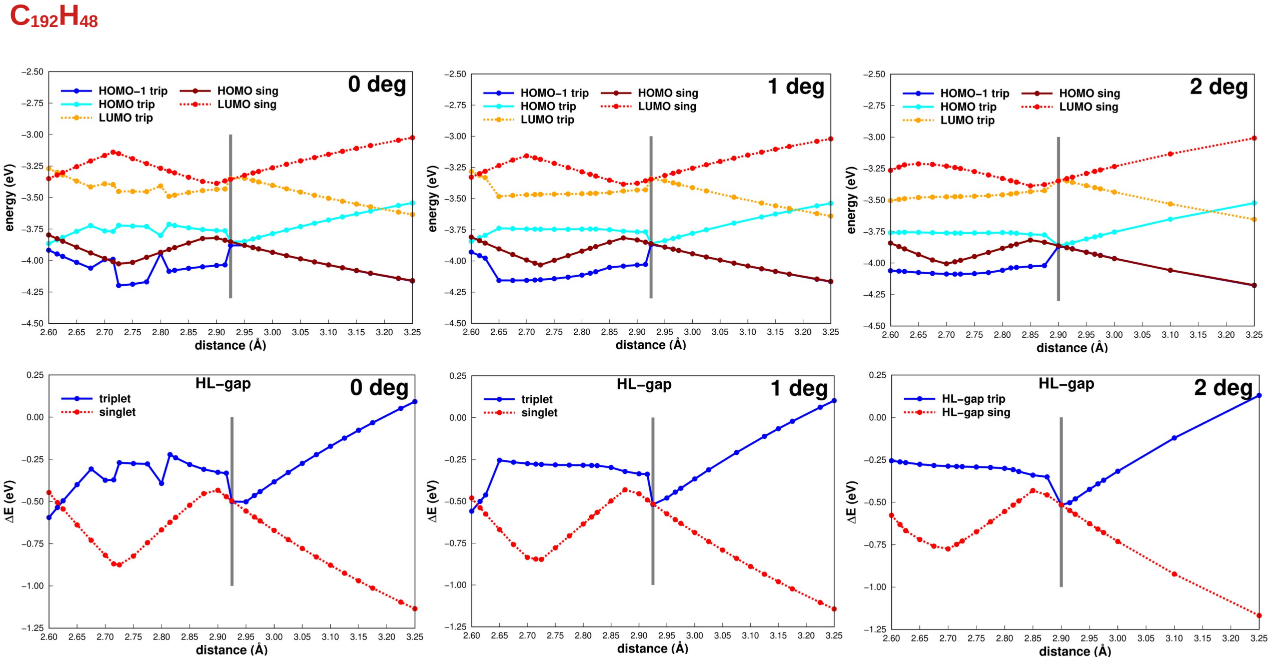

Figure S5: Orbital energies (HOMO-1, HOMO, and LUMO) and HOMO-LUMO energy gap for $C_{192}H_{48}$ graphene nanoflake.

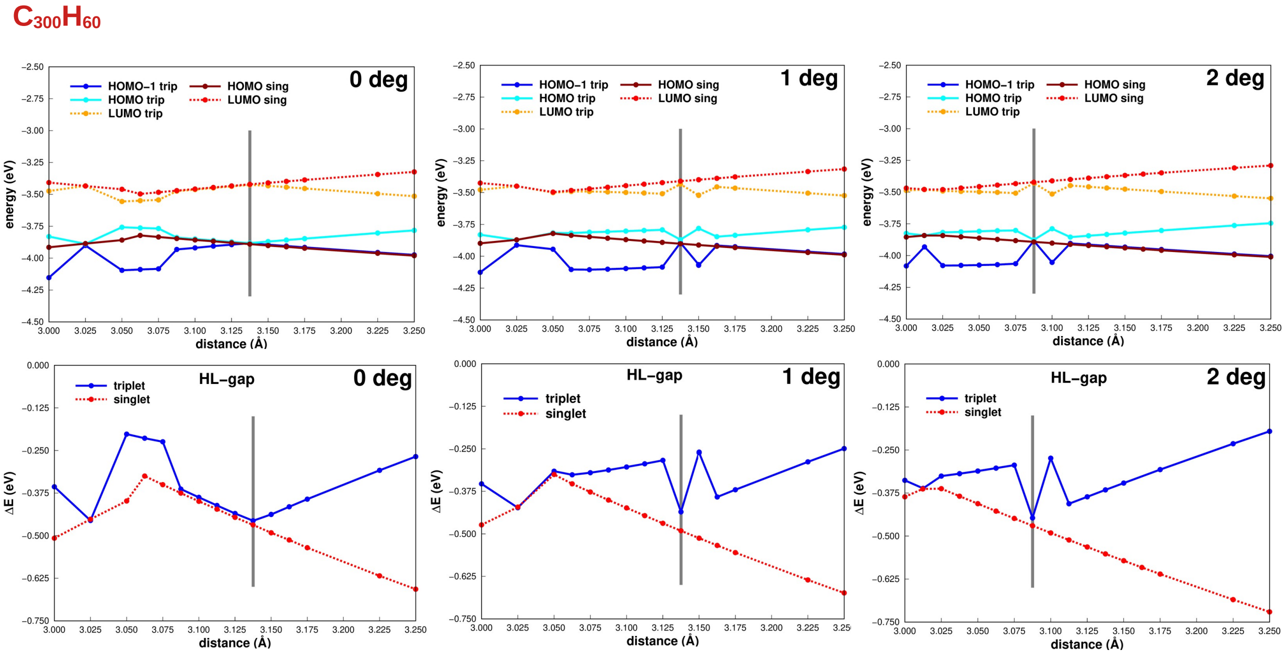

Figure S6: Orbital energies (HOMO-1, HOMO, and LUMO) and HOMO-LUMO energy gap for $C_{300}H_{60}$ graphene nanoflake.

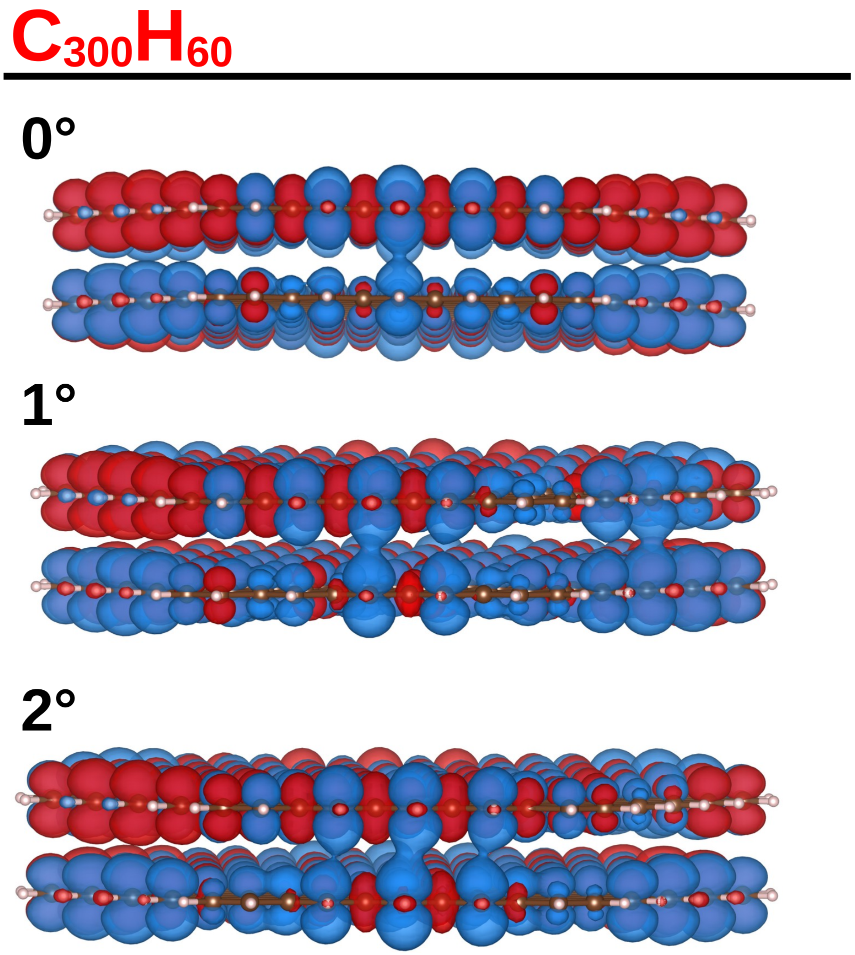

Figure S7: Side view of the spin-density of the $C_{300}H_{60}$ nanoflakes for 0°, 1°, and 2° twisted angles.
