# Supplementary material for: Magnetic Moment and Spin-State Transitions in Twisted Graphene Nanostructures
Source: J Phys Chem Lett. 2025 Feb 18;16(8):1994–2000. doi: 10.1021/acs.jpclett.4c03542 (PMC11873933; doi:10.1021/acs.jpclett.4c03542)
Supplement: Supplementary file 2 — jz4c03542_si_002.pdf [file jz4c03542_si_002.pdf]

jz-2024-03542v.R1

Name: Peer Review Information for "Magnetic Moment and Spin-State Transitions in Twisted Graphene Nanostructures"

First Round of Reviewer Comments

Reviewer: 1

Comments to the Author

The authors systematically investigate the emergence of magnetic moment in twisted bilayer graphene nanoflakes. Their results highlight the importance of repulsive interaction induced by interlayer strain in inducting magnetism. The representations are compelling and provide valuable insights into this field. However, I have the following comments that need to be addressed before publication:

1. The author only considers the out-of-plane strains on the magnetic properties, but the influence of in-plane strain should be discussed.

2. Following the first question, can the author compare their result with fully relaxed one (the atoms can only be relaxed in the in-plane direction)? This comparison would provide a more complete understanding of strain effects.

3. The authors select 1°, 2°, 3°, 7°, 30° twisted angles in their study but do not provide reasons. Can the authors explain for these choices?

4. In the left panel of line 50, page 2, the authors conclude that their work will be helpful to “understanding why the magic angle in twisted bilayer graphene is approximately 1°”. However, it seems the manuscript does not provide clear evidence or discussion supporting this claim.

5. In the left panel of line 54, page 2, the author mention “C192H48, C300H60, and C432H72, as shown in Figures 2-(a-i) and in Supplementary Material (SM)”. However, the configuration for C432H72 is missing from both Figure 2 and the SM.

6. In the left panel of line 39, page 5, the authors decompose the total energy into different part, i.e. correlation energy, exchange energy, kinetic energy and exchange and correlation energy. They discussed how their interplay will result in triplet ground state. This perspective sound interesting but seem superficial. Can the authors give deeper explanations how they related to the emergence of magnetic moment?

7. In Figure 5, the authors suggest that magnetic moments between intralayer and interlayer nearest carbon atoms are antiferromagnetically coupled. Is this the only observed magnetic ordering? Have the authors compared the total energies of different magnetic orderings to confirm this?

8. The authors claim “The spin-up density is predominant in the nanoflakes”, but do not provide detailed evidence. I think the authors can provide total magnetic moment against twisted angle.

9. As magnetic moment emerges in the repulsive region, this will increase the possibility of out-of-plane orbital overlap, e.g. C-pz. Could this overlap contribute to the emergence of the magnetic moment? Moreover, does the magnetic moment originate from out-of-plane pz or in-plane sp2 orbitals? I think the authors should give a side view of the spin density to clarify this.

10. Figures or tables from the supporting information should be mentioned successively in the main text. Currently, only Figure S3/S4/S5 is referenced, which needs to be addressed.

11. In Table 1, the authors mention obtaining interlayer distances through polynomial fitting but provide no details. Including the fitting formula and a comparison between the fitting curve and calculated data would enhance clarity.

12. Some editing advices:

a. In Figure caption of Figure 3, “Potential energy curve” should be “Potential energy curves”; “for AA stacking for C192H48 structure” should be “the AA stacking of C192H48”. The same revision applies to the Figure caption of Figure 4.

b. In the left panel of line 7/8, page 5, “respectively” should be inserted at the end of the sentence.

c. In the right panel of line 7, “The HOMO and the HOMO-1 and LUMO of the singlet and triplet assume the same values” should be “The HOMO and the HOMO-1 of the triplet, and HOMO of singlet assume the same values”.

Reviewer: 2

Comments to the Author

The authors predicted by DFT calculations that structural

and electronic conditions govern the magnetic properties in twisted graphene bilayers.

The repulsive interaction between the layers in the AA region proves to be a fundamental ingredient

to the emergency of the magnetic moment from the singlet-triplet spin state transition.

Furthermore, the size of the AA stacking region is directly connected with the distance

at which the spin state transition occurs, i.e., a smaller AA region implies a shorter interlayer distance. I think the results are moderate interesting, which may just reach the edge of the criterion for JPCL, and I do not have much suggestions for further modifications. The AA stacking is much higher in energy compared with AB stacking, and the configurations in Fig. 2 are highly unstable. Even twisted graphene bilayer can be stabilized, the small twisted graphene flakes may automatically become untwisted, and the authors may check this point.

Author's Response to Peer Review Comments:

Dear Editor,

Please find enclosed the revised version of the manuscript entitled: "***Magnetic Moment and Spin-State Transitions in Twisted Graphene Nanostructures***" that we have submitted for publication in **The Journal of Physical Chemistry Letters**. We sincerely thank the reviewers for their positive assessment of our work and for their valuable comments. We believe their suggestions have helped to enhance both the quality and presentation of our manuscript. The manuscript has been revised accordingly, with all changes highlighted in red color in the main text. Below, we also provide a point-by-point response to each of the reviewers' questions and comments.

We sincerely hope that you will judge our revised manuscript to be suitable for publication in **The Journal of Physical Chemistry Letters**.

Reviewer: 1

Recommendation: This paper may be publishable, but major revision is needed; I would like to be invited to review any future revision.

Comments:

The authors systematically investigate the emergence of magnetic moment in twisted bilayer graphene nanoflakes. Their results highlight the importance of repulsive interaction induced by interlayer strain in inducting magnetism. The representations are compelling and provide valuable insights into this field. However, I have the following comments that need to be addressed before publication:

Reviewer 1: 1) The author only considers the out-of-plane strains on the magnetic properties, but the influence of in-plane strain should be discussed.

> **Authors:** We thank the reviewer for raising this point. Indeed, the investigation of the effect of the in-plane strain on the magnetic properties of twisted graphene bilayer is an interesting topic for future investigations. In this regard, a previous study [PRB 96 (2017) 115428] has investigated the behavior of magnetic properties under the in-plane uniaxial strain of the AB and AA bilayer graphene nanoflakes. The conclusion is that the strain modifies the magnetic moments providing a complex ordering that has a mix of ferromagnetic and antiferromagnetic couplings between the two layers. This behavior is similar to the one observed in our results, which is now discussed in the manuscript as follows:
>
> "This behavior is similar to that observed for graphene nanoflakes with AA and AB staking under in-plane uniaxial strain [57], where the strain modifies the magnetic moments providing a complex ordering that has a mix of ferromagnetic and antiferromagnetic couplings between the two layers."

Reviewer 1: 2) Following the first question, can the author compare their result with fully relaxed one (the atoms can only be relaxed in the in-plane direction)? This comparison would provide a more complete understanding of strain effects.

> **Authors:** Due to the high computational cost involved in the calculation, the fully relaxed in-plane optimization could make the calculations unfeasible. However, the model used in the present work was able to describe the most relevant qualitative aspects of the interplay between the magnetic properties and the out-of-plane strain.

Reviewer 1: 3) The authors select 1°, 2°, 3°, 7°, 30° twisted angles in their study but do not provide reasons. Can the authors explain for these choices?

> **Authors:** The choice of the small angles (0°-2°) is directly motivated by the experimental achievements, where the observed Moiré pattern arises for angles around 1°. Additionally, due to the symmetry of the systems, angles higher than 30° will provide the same results.

Reviewer 1: 4) In the left panel of line 50, page 2, the authors conclude that their work will be helpful to "understanding why the magic angle in twisted bilayer graphene is approximately 1°". However, it seems the manuscript does not provide clear evidence or discussion supporting this claim.

Authors: Thank you for given us the opportunity to further clarify this point. The last paragraph of the manuscript now reads:

"The repulsive interaction between the layers in the AA region proves to be a fundamental ingredient to the emergency of the magnetic moment from the singlet-triplet spin state transition. Furthermore, the size of the AA stacking region is directly connected with the distance at which the spin state transition occurs, i.e., a smaller AA region implies a shorter interlayer distance. Therefore, selecting the appropriate twist angle is crucial for generating a sufficiently large AA stacking region, allowing the spin state transition to occur at a distance similar to that induced by the AB stacking. Thus, based on our findings, the twisted angle close to 1° fulfills the necessary conditions for the emergency of the magnetic moment."

Reviewer 1: 5) In the left panel of line 54, page 2, the author mention "C192H48, C300H60, and C432H72, as shown in Figures 2-(a-i) and in Supplementary Material (SM)". However, the configuration for C432H72 is missing from both Figure 2 and the SM.

Authors: Thank you. The C432H72 figure is now in the SM.

Reviewer 1: 6) In the left panel of line 39, page 5, the authors decompose the total energy into different part, i.e. correlation energy, exchange energy, kinetic energy and exchange and correlation energy. They discussed how their interplay will result in triplet ground state. This perspective sound interesting but seem superficial. Can the authors give deeper explanations how they related to the emergence of magnetic moment?

Authors: Thank you for your insightful question. It is important to highlight that the emergence of the magnetic moment is not solely explained by the interplay of the DFT total energy components. As discussed in [PhysRevLett.119.107201], at a mean-field level, spontaneous magnetization of two different types may develop for small enough twist angles ($\theta \lesssim 2^{\circ}$) as a consequence of the moiré pattern in the system. This effect arises due to the high local density of states generated close to neutrality at moiré regions with AA stacking, which triggers a Stoner instability when electrons interact. In this context, our goal was to verify whether the contributions to the total energy could, even qualitatively, indicate this electron interaction. Specifically, we aimed to illustrate the increase in electronic correlation in the repulsive region, thereby linking the DFT total energy decomposition to the underlying physical mechanisms driving spin polarization and magnetic moment formation. Specifically, Figures S5 and S6 of the SM show that, in the repulsive region, the reduction (in magnitude) of

the electronic kinetic energy and the increase (in magnitude) of the exchange-correlation energy are indicators of enhanced electronic instability.

We have added the reference [PhysRevLett.119.107201] to the manuscript and revised the discussion as follows:

In the DFT framework, the total energy decomposition can offer insights into the physical mechanisms driving spin polarization and magnetic moment formation. As illustrated in Figures S5 and S6 of the SM (for the $C_{300}H_{60}$ and $C_{432}H_{72}$ systems, respectively), the reduction (in magnitude) of the electronic kinetic energy and the increase (in magnitude) of the exchange-correlation energy near the minimum in ΔE serve as indicators of enhanced electronic correlation in the repulsive region, which triggers a Stoner instability when electrons interact, aligning with the mechanisms leading to spin polarization as discussed in[53]. Notably, these changes are less pronounced in the triplet energy components, as also shown in Figures S4 and S5.

Reviewer 1: 7) In Figure 5, the authors suggest that magnetic moments between intralayer and interlayer nearest carbon atoms are antiferromagnetically coupled. Is this the only observed magnetic ordering? Have the authors compared the total energies of different magnetic orderings to confirm this?

Authors: Thank you for the questions. The antiferromagnetic coupling between the sublattice A and B is expected for the close-shell monolayer systems, as discussed in reference [Phys. Rev. Lett. 2007, 99, 177204]. For open-shell monolayer systems, this coupling is a ferrimagnetic one [Carbon 2023, 213, 118186.]. This coupling, antiferromagnetic in the close-shell monolayer, is broken by the repulsive interaction between the two monolayers, providing a ferromagnetic coupling (see Figure 5 of the manuscript). Regarding comparing the energies of various magnetic orderings, the model employed in this research does not allow for comparing the energies of different couplings between sublattices A and B.

Reviewer 1: 8) The authors claim "The spin-up density is predominant in the nanoflakes", but do not provide detailed evidence. I think the authors can provide total magnetic moment against twisted angle.

Authors: Thank you for the question. By definition (and by a limitation of the level of theory applied), the semi-occupied orbitals are fulfilled by spin alpha electrons, which keep the magnetic moment constant. Therefore, the spin-up density should be prominent for the triplet spin state. By Figure 5 of the manuscript, it is possible to observe that the blue color (up) is dominant, mostly on the edge of the systems.

Reviewer 1: 9) As magnetic moment emerges in the repulsive region, this will increase the possibility of out-of-plane orbital overlap, e.g. C-pz. Could this overlap contribute to the emergence of the magnetic moment? Moreover, does the magnetic moment originate from out-of-plane pz or in-plane sp2 orbitals? I think the authors should give a side view of the spin density to clarify this.

Authors: Thanks for raising this point. In fact, in the repulsive region the out-of-plane orbital overlap is maximized, favoring π-π interactions. The side view of the spin density is now gathered in SM.

Reviewer 1: 10) Figures or tables from the supporting information should be mentioned successively in the main text. Currently, only Figure S3/S4/S5 is referenced, which needs to be addressed.

Authors: Thank you. The figures are now mentioned in the manuscript.

Reviewer 1: 11) In Table 1, the authors mention obtaining interlayer distances through polynomial fitting but provide no details. Including the fitting formula and a comparison between the fitting curve and calculated data would enhance clarity.

Authors: Thank you for the point. The fitting was performed from a sixth-order polynomial equation using the points close to the minimum of the Potential Energy Curve. This explanation was added in Table 1 of the manuscript.

Reviewer 1: 12) Some editing advices:

a. In Figure caption of Figure 3, "Potential energy curve" should be "Potential energy curves"; "for AA stacking for C192H48 structure" should be "the AA stacking of C192H48".
The same revision applies to the Figure caption of Figure 4.

Authors: We have changed the captions of Figures accordingly. Thank you.

b. In the left panel of line 7/8, page 5, "respectively" should be inserted at the end of the sentence.

Authors: Done. Thank you.

c. In the right panel of line 7, "The HOMO and the HOMO-1 and LUMO of the singlet and triplet assume the same values" should be "The HOMO and the HOMO-1 of the triplet, and HOMO of singlet assume the same values".

Authors: Thank you. The sentence was corrected.

Reviewer: 2

Recommendation: This paper is probably publishable, but major revision is needed; I do not need to see future revisions.

Comments:

The authors predicted by DFT calculations that structural and electronic conditions govern the magnetic properties in twisted graphene bilayers. The repulsive interaction between the layers in the AA region proves to be a fundamental ingredient to the emergency of the magnetic moment from the singlet-triplet spin state transition. Furthermore, the size of the AA stacking region is directly connected with the distance at which the spin state transition occurs, i.e., a smaller AA region implies a shorter interlayer distance. I think the results are moderate interesting, which may just reach the edge of the criterion for JPCL, and I do not have much suggestions for further modifications. The AA stacking is much higher in energy compared with AB stacking, and the configurations in Fig. 2 are highly unstable. Even twisted graphene bilayer can be stabilized, the small twisted graphene flakes may automatically become untwisted, and the authors may check this point.

> **Authors:** We appreciate the positive assessment of our findings. We believe our study provides valuable insights into the relationship between structural and electronic conditions in twisted graphene nanostructure bilayers and the emergence of magnetic moments.

jz-2024-03542v.R2

Name: Peer Review Information for "Magnetic Moment and Spin-State Transitions in Twisted Graphene Nanostructures"

Second Round of Reviewer Comments

Reviewer: 1

Comments to the Author

I am satisfied with the revisions the authors made.

Reviewer: 2

Comments to the Author

The authors ignored my concern:

The AA stacking is much higher in energy compared with AB stacking, and the configurations in Fig. 2 are highly unstable. Even twisted graphene bilayer can be stabilized, the small twisted graphene flakes may automatically become untwisted, and the authors may check this point.

Author's Response to Peer Review Comments:

We sincerely thank the Editor and reviewers for their thorough evaluation of our manuscript and their constructive comments and suggestions. The feedback provided has been invaluable in improving the quality of our work. We appreciate the time and effort dedicated to reviewing our manuscript and for recognizing its significance.

Reviewer: 1

Recommendation: This paper represents a significant new contribution and should be published as is.

Comments:

I am satisfied with the revisions the authors made.

Author: We thank Reviewer 1 for their positive feedback and for recognizing the significance of our work.

Reviewer: 2

Recommendation: This paper is publishable subject to minor revisions noted. Further review is not needed.

Comments:

The authors ignored my concern: The AA stacking is much higher in energy compared with AB stacking, and the configurations in Fig. 2 are highly unstable. Even twisted graphene bilayer can be stabilized, the small twisted graphene flakes may automatically become untwisted, and the authors may check this point.

Authors: The reviewer raises an important point regarding the higher energy and instability of AA stacking compared to AB stacking which we should have addressed in our first reply. We deeply apologize and submit below our reply for consideration:

It is well established in the literature that AA stacking is less stable than AB stacking in bulk graphene, as demonstrated in several studies and noted in the manuscript. In the context of graphene-based nanostructures, particularly twisted bilayers, and small flakes, the energetic differences can be less pronounced due to edge effects and external factors such as strain or substrate interactions. For example, bilayer graphene with precise control over the twist angle has been experimentally prepared, predominantly by folding graphene or using aligned transfer of single-crystalline graphene [Chem. Mater. 2020, 32, 24, 10357–10364]. Additionally, the literature shows that for small graphene bilayers or twisted bilayers, even configurations with higher energy than bulk AB stacking can exhibit unique electronic properties, including the emergence of magnetism and topological states [Nano Lett. 2022, 22, 15, 6186–6193; Proc. Natl. Acad. Sci. U.S.A. 2011, 108, 30, 12233–12237].

We emphasize that the primary goal of the present work is to use graphene nanoflakes as a model system to investigate the emergence of magnetic moments in the AA-stacked regions of twisted graphene bilayers. Our aim is to shed light on the fundamental mechanisms governing spin-state transitions and magnetic properties in these regions. However, the AA-stacked configurations in Figure 2 correspond to stable local minima for the nanostructures under consideration, as supported by the potential energy curves in Figures 3 and 4. Thus, these structures represent metastable states that can persist under specific conditions.

To estimate the out-of-plane pressure necessary to keep the system in the triplet spin state, we added the following paragraph in the manuscript and also provided a full explanation of the pressure calculation in SM.

"Although AA stacking is energetically less favorable than AB stacking, our calculations indicate that the structure remains in a local metastable state, as shown in Figures 3 and 4. Furthermore, the required out-of-plane pressure to maintain the necessary distance for the triplet spin-state in the C300H60 system at 1° is moderate, around -5.56 GPa, as discussed in SM. Our results for nanoflakes agree with experiments on moiré superlattices of graphene encapsulated in h-BN [53-55], where it was shown that applying pressure increases the magic angle in the phase transition. This suggests that the configuration studied here could, in principle, be stabilized under experimentally feasible conditions."
